# The use of direct oral anticoagulants in children with congenital and acquired heart disease

**DOI:** 10.3389/fcvm.2026.1920897

**Published:** 2026-08-07

**Authors:** Eman Abdelghani, Mehul Thakkar, Jessica Berthiaume, Sanjay Ahuja

**Affiliations:** Department of Pediatric Hematology, Indiana Hemophilia and Thrombosis Center, Innovative Hematology Inc., Indianapolis, IN, United States

**Keywords:** anticoagulation, children, congenital heart disease, direct oral anticoagulant, thrombosis

## Abstract

Direct oral anticoagulants (DOACs), including the direct thrombin inhibitor, dabigatran, and factor Xa inhibitors (rivaroxaban, apixaban, and edoxaban), represent a significant advancement in pediatric anticoagulation therapy. Following U.S. FDA approvals for dabigatran and rivaroxaban in 2021 and apixaban in 2025, DOACs are now available for venous thromboembolism (VTE) prevention and treatment in pediatric patients; edoxaban has not yet received pediatric approval. Children with congenital and acquired heart disease (CAHD) constitute a particularly high-risk population for thromboembolic complications due to altered hemodynamics, prosthetic materials, and surgical interventions, necessitating effective anticoagulation strategies. This review summarizes current evidence regarding DOAC use in pediatric CAHD, evaluates safety and efficacy data from recent clinical trials, and highlights key knowledge gaps and future research priorities.

## 1 Introduction

Pediatric heart disease, encompassing both congenital and acquired heart disease (CAHD), represents a major public health challenge. Pediatric acquired heart disease is less common, but global data suggests rheumatic heart and Kawasaki disease (more prevalent in high income countries) account for most cases (1, 2). Congenital heart disease (CHD) affects approximately 1%–1.2% of livebirths and is the most common congenital condition in childhood, with an estimated prevalence of 13 per 1,000 children (3). Advances in diagnosis, medical management, and surgical interventions have dramatically improved outcomes, with more than 90% of children with CHD now surviving into adulthood (4). Additionally, recent analyses indicate that the global prevalence of CHD in children under 5 years old exceeded 4.18 million cases in 2021, reflecting a 3.4% increase in absolute case numbers since 1990 (4, 5) and underscoring the need for effective clinical treatments to manage the disease.

Thromboembolic events (TE) are among the most serious complications of CAHD and contribute substantially to morbidity and mortality. Children with shunt-dependent single ventricles, Fontan circulation, Kawasaki disease with giant coronary aneurysm, cardiomyopathy, and those with central venous lines (CVL) are at particularly high risk (6, 7). The incidence of thrombotic complications varies widely according to the underlying diagnosis and patient population. While venous thromboembolism (VTE) is the most frequent thrombotic manifestation, arterial thrombotic events like myocardial infarction and ischemic stroke are observed less frequently but remain clinically significant contributors to morbidity and mortality (8, 9). In a large children's hospital-acquired thrombosis registry (CHAT), CHD was present in ∼27% of children with VTE (10). In children undergoing cardiac surgery, 3.6%–11% develop post-operative thrombosis which can be life-threatening (9, 11–13). Risk of recurrent TE is also high; up to 25% within the first year of index VTE (9). Children with CHD also account for a disproportionate share of pediatric stroke, representing an estimated 30% of all childhood strokes, and a 19-fold increased risk of stroke compared to the general population (14). These observations underscore the critical role of thromboprophylaxis in this vulnerable population.

Thrombosis prevention in pediatric CAHD remains challenging. Historically, antithrombotic strategies relied heavily on vitamin K antagonists (VKAs), particularly warfarin, despite the considerable limitations associated with pediatric use. Frequent laboratory monitoring, narrow therapeutic windows, numerous drug-drug and drug-food interactions, and dietary restrictions can complicate treatment adherence and negatively affect quality of life for patients and families, in addition to long-term bone density reduction with increased risk of fracture. Furthermore, VKAs lack a commercially available pediatric liquid formulation (15, 16). Although direct oral anticoagulants (DOACs), which directly inhibit thrombin (dabigatran) or factor Xa (apixaban, rivaroxaban, edoxaban), have transformed thromboprophylaxis in adults due to predictable pharmacokinetics and ease of administration, evidence supporting their use in pediatric patients has emerged only recently (17–21).

**Figure 1:**
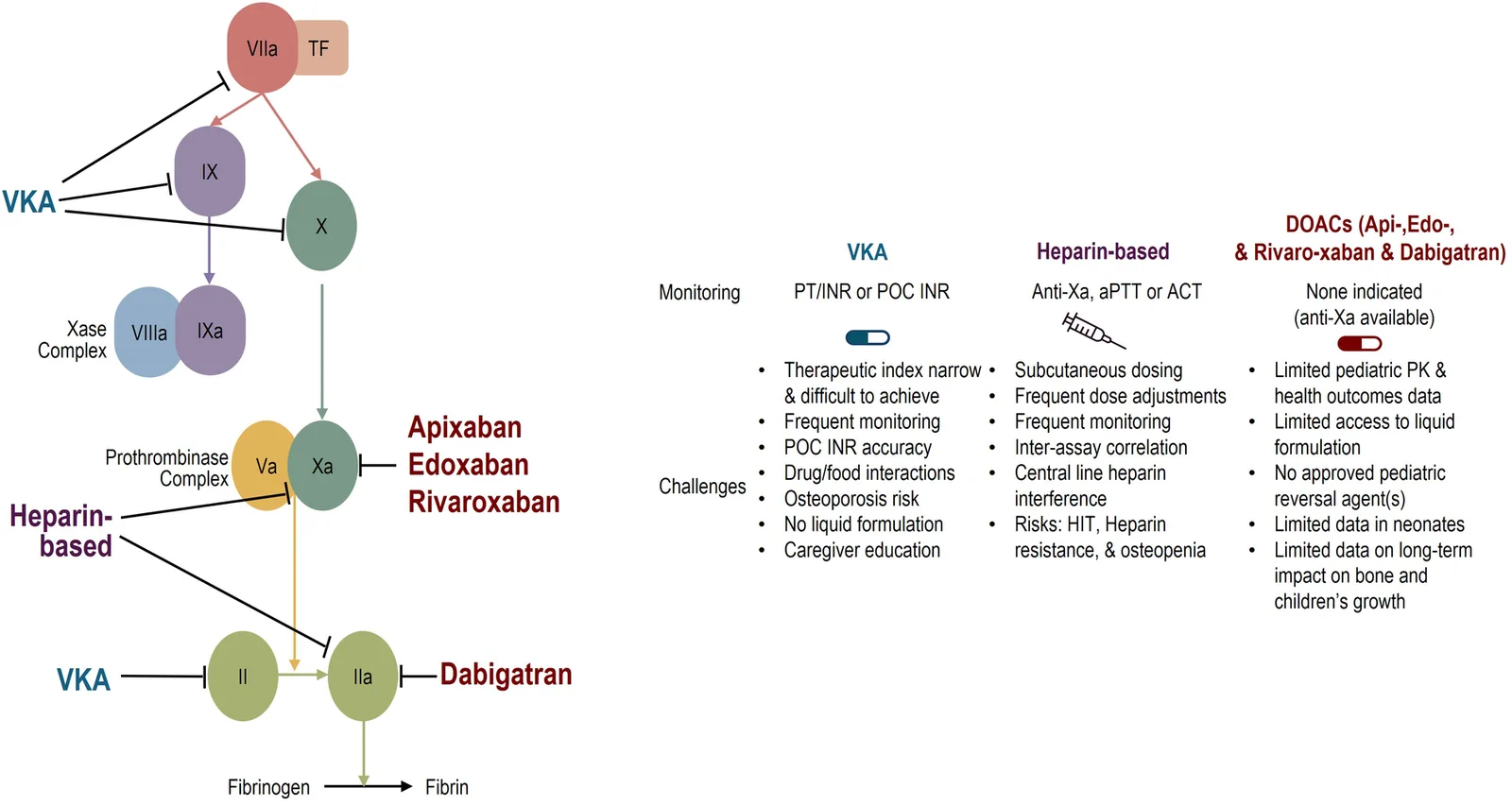
The coagulation cascade, anticoagulant targets, laboratory monitoring assays, and associated challenges in pediatric heart disease. Vitamin K antagonists (VKAs) inhibit synthesis of factors II, VII, IX, and X; heparin- based anticoagulation include unfractionated heparin (UFH) and low-molecular-weight heparin (LMWH) which both potentiate antithrombin-mediated inhibition of thrombin and factor Xa; and direct oral anticoagulants (DOACs) directly inhibit either thrombin (dabigatran) or factor Xa (apixaban, edoxaban, rivaroxaban). Key monitoring assays and pediatric-specific are outlined for each drug class. PT, prothrombin time; INR, International Normalized Ratio; POC, point-of-care, aPTT, activated partial thromboplastin time; ACT, activated clotting time; HIT, heparin induced thrombocytopenia.

Dabigatran received FDA approval in 2021 based on the DIVERSITY trial (18) and is indicated for treatment and secondary prophylaxis of VTE in children (3 months to 18 years) after at least 5 days of parenteral heparin-based anticoagulation. Rivaroxaban approval in 2021 was initially supported by the EINSTEIN-Jr study (17) for treatment and secondary prophylaxis of VTE in pediatric patients from birth to <18 years after at least 5 days of parenteral anticoagulant, and then in 2024 following the UNIVERSE study (19) for thromboprophylaxis in children with CHD >2 years of age following the Fontan procedure. Apixaban was just recently approved in 2025 for VTE treatment and secondary thromboprophylaxis for children at birth and older after at least 5 days of initial anticoagulant treatment. Edoxaban has not yet received FDA approval, despite favorable trial data in pediatric populations. For current prescribing and use information, readers are referred to the online FDA database (https://open.fda.gov/fdalabels/). Overall DOACs have proven safe and efficacious for clinic use in children, however, they have limited approvals in pediatric CAHD. Figure 1 provides an integrated overview of the coagulation cascade and commonly used anticoagulants in children with heart disease and principal challenges related to each class of anticoagulants.

The increasing availability of pediatric-specific data and regulatory approvals has generated growing interest in the role of DOACs across the spectrum of pediatric CAHD. However, important questions remain regarding patient selection, optimal dosing strategies, comparative effectiveness, safety, and the application of these agents to specific high-risk cardiac populations. This review summarizes the current evidence supporting the use of DOACs in pediatric CAHD, highlights practical considerations for clinicians, and identifies key areas for future investigation as the field continues to evolve.

A comprehensive literature search was conducted using PubMed, Embase, and Google Scholar databases from inception through June 2025. Search terms included combinations of ‘direct oral anticoagulants,’ ‘DOACs,’ ‘apixaban,’ ‘rivaroxaban,’ ‘dabigatran,’ ‘edoxaban,’ ‘pediatric,’ ‘children,’ ‘congenital heart disease,’ ‘acquired heart disease,’ ‘Fontan,’ ‘thromboprophylaxis,’ ‘anticoagulation,’ and ‘venous thromboembolism.’ Original research articles, clinical trials, systematic reviews, meta-analyses, case series, and clinical practice guidelines published in English were included. Google Scholar was used as a supplementary resource to capture additional relevant publications not indexed in traditional databases. Reference lists of identified articles were manually reviewed to identify further pertinent studies. As this is a narrative review, a formal systematic review methodology was not employed.

## 2 Pathophysiology and risk factors of thromboembolism in pediatric CAHD

The pathophysiology of thromboembolism in CAHD is complex and multifactorial, arising from the interplay between components of Virchow's triad: abnormal blood flow, endothelial injury, and hypercoagulability (22). The relative contribution of each to thrombosis risk varies according to the underlying cardiac lesion, surgical intervention, patient age, and stage of treatment.

Blood flow abnormalities are often noted in conditions such as Fontan and Glenn circulations where velocity is reduced, as well as in dilated cardiomyopathy or heart failure where cardiac output is greatly diminished (22). Vessel wall injury and endothelial dysfunction often result from surgical interventions, CVL placement, the inflammatory response to cardiopulmonary bypass, and implantation of thrombogenic prosthetic materials like valves, conduits, stents, and surgical shunts (6, 23, 24). A state of hypercoagulability in CAHD is fostered by post-operative inflammation and developmental hemostasis, marked by coagulation factor imbalances such as reduced antithrombin levels resulting in relative heparin resistance in young children (6, 25, 26). Additionally, chronic hypoxemia in cyanotic heart disease further augments hypercoagulability by triggering secondary erythrocytosis and hyperviscosity, leading to a significantly increased risk of thrombosis, paradoxical embolism, and ischemic stroke (27, 28).

Among congenital heart lesions, single-ventricle physiology is associated with the greatest thrombotic risk. The cumulative burden of altered hemodynamics, repeated surgical interventions, endothelial injury, and coagulation abnormalities contribute to persistently elevated thromboembolic risk throughout staged palliation. Reported incidences of thrombosis approach 40% following stage I palliation, 28–29.3% after the bidirectional Glenn procedure, and a cumulative incidence of 21.2% between stage I and stage II palliation in the Pediatric Heart Network Single Ventricle Trial (9, 29, 30). Large multicenter studies have further demonstrated that extremity venous thrombosis is the most common TE complication following pediatric cardiac surgery, accounting for approximately 94% of TE in children with CAHD (9) and 88% in those with CHD (31), reflecting the frequent use of central venous access and prolonged hospitalization in these populations.

CVL placement is the most significant modifiable risk factor for hospital-acquired thrombosis in pediatric CAHD, increasing the risk of VTE more than ninefold (odds ratio of 9.14; 95% CI, 3.38–24.72) (31). Other risk factors associated with thrombosis in patients with CAHD, include younger age (<12 months), prolonged cardiopulmonary bypass time, greater surgical complexity, single-ventricle physiology, prior thrombotic events, ICU admission, mechanical circulatory support, and prior hospitalization (31, 32). Recognition of these patient- and procedure-specific risk factors is essential for individualized thromboprophylaxis and can help inform how to implement DOACs in pediatric CAHD.

## 3 Current evidence on the use of DOACs in children with CAHD

### 3.1 Current clinical practice guidelines

Clinical practice guidelines in CAHD are rapidly evolving as pediatric-specific evidence supporting DOACs emerges. Most recommendations were developed prior to pivotal pediatric DOAC trials thus largely reflect the historical reliance on VKAs, particularly warfarin.

Early guidance was understandably conservative. The Pediatric and Congenital Electrophysiology Society (PACES)/Heart Rhythm Society (HRS) 2014 Expert Consensus Statement assigned only a weak Class IIb recommendation for DOAC use in adults with simple CHD without significant valvular disease, reflecting the limited evidence available (33). Similarly, the 2015 American Heart Association (AHA) Scientific Statement on CHD in older adults noted that DOACs have not been adequately studied in adults with CHD and recommended warfarin as the preferred anticoagulant for patients with sustained atrial fibrillation (34).

Pediatric guidance has likewise been limited. The American Society of Hematology (ASH) 2018 guidelines on management of VTE in pediatrics focused on VKAs, and DOACs were considered out of the scope because large-scale pediatric DOAC trials were incomplete (35). More recent guidelines acknowledge the growing interest in DOACs while highlighting the lack of robust evidence in high-risk CHD populations. The 2021 AHA guidelines for stroke prevention maintain warfarin as the preferred agent for secondary prevention in patients with Fontan circulation, citing the paucity of safety and efficacy data for DOACs, particularly in the setting of Fontan-associated hepatic dysfunction (36). Likewise, the 2025 ACC-AHA-HRS-ISACHD-SCAI guidelines provide recommendations on anticoagulation and antiplatelet therapies for adults with CHD without specific guidance on DOACs (37).

Recognizing these knowledge gaps, the 2026 ASH/ISTH guidelines for anticoagulant prophylaxis in pediatric patients at risk of VTE specifically acknowledge that antithrombotic management in children with cardiac disease represents a distinct and complex clinical area that warrants dedicated evidence-based guidance beyond current recommendations (38).

### 3.2 Pediatric DOAC clinical trials in cardiac populations

Given the lack of consensus guidelines on the use of DOACs in pediatric cardiac patients, several landmark clinical trials have evaluated the pharmacokinetics (PK), safety, and efficacy of DOACs in this high-risk population. The principal studies are summarized below:

#### 3.2.1 Safety of apiXaban on pediatric heart disease on the preventioN of embolism (SAXOPHONE trial published in 2023)

This was a randomized, open label, multicenter, phase 2 trial comparing apixaban to standard of care (SOC) including VKA or low molecular weight heparin (LMWH) in children with CAHD requiring thromboprophylaxis (21). Apixaban PK data using weight-based adjusted doing was consistent with successful adult dosing. Please refer to Table 1 for summary on efficacy and safety outcomes. It was concluded that apixaban is safe and well-tolerated in children with CAHD requiring chronic thromboprophylaxis. Limited data was collected regarding bone density and quality of life, and thus interpretation was limited due to small sample size.

**Table 1:** Summary of pivotal clinical trials evaluating the safety and efficacy of DOACs in pediatric heart disease.

Trial	DOAC tested	Mean duration of intervention	Population	Age range	Efficacy outcomes	Safety outcomes; bleeding events
SAXOPHONE	Apixaban (*n* = 123) vs SOC (*n* = 36)	330 days (mean) in apixaban vs 344 days in SOC arm Follow-up duration: up to 12 months	CAHD requiring thromboprophylaxis (majority enrolled had Fontan physiology and Kawasaki disease with giant coronary artery aneurysm)	28 days to <18 years (majority enrolled were 2–12 years)	TE: 0	Major and CRNMB: 1.8 per 100 person-years in the apixaban arm vs 6.8 in SOC arm. Minor: Higher rate on apixaban consisting mainly of epistaxis, contusion, hematoma, and 2 participants with menstrual-related bleeding
ENNOBLE-ATE	Edoxaban (*n* = 109) vs SOC (*n* = 58)	Participants received randomized treatment for 3 months *(main study period)* 147 children transitioned into an open-label edoxaban *extension arm* for up to 9 additional months	CAHD requiring thromboprophylaxis [stratified by cardiac diagnosis, with Fontan surgery (44%) and Kawasaki disease (22%) being the most common indications]	Gestational age of ≥ 38 weeks to <18 years (majority enrolled were 2–12 years)	Main study period outcomes: TE: 0 edoxaban vs 1.7% in SOC Extension period outcomes (edoxaban only): TE: 2.1% (one stroke and two coronary artery thromboses in Kawasaki disease patients)	Main study period outcomes: Major: 0 CRNMB: 0.9% edoxaban vs 1.7% SOC Extension period outcomes (edoxaban only): Major: 1.4% (primary hemorrhagic stroke and abdominal bleed following injury-related liver laceration)
UNIVERSE	Rivaroxaban (*n* = 66) vs ASA (*n* = 34)	12 months Follow-up duration: 12 months	Single-ventricle physiology who completed Fontan procedure within 4 months of enrollment	2–8 years	TE: 2% rivaroxaban vs 9% ASA	Major: 1.5% rivaroxaban vs 0% ASA CRNMB: 6% rivaroxaban vs 9% ASA Minor: 33% rivaroxaban vs 35% ASA
DIVERSITY post-hoc CHD subgroup analysis	Dabigatran (*n* = 21) vs SOC (*N* = 27)	3 months Follow-up duration: 12 months	VTE treatment and secondary prevention in CHD (70.8% had CVL-related thrombosis)	0–17 years (median age 1 year in dabigatran vs 3 years in SOC)	primary (complete thrombus resolution, no VTE recurrence, and no VTE-related deaths): 81.0% on dabigatran VS 59.3% on SOC	Major: 0 CRNMB: 0 Minor: 14.3% dabigatran vs 3.7% SOC

DOAC, direct oral anticoagulant; SOC, standard of care; CAHD, congenital and acquired heart disease; ASA, aspirin; TE, thrombotic events; CRNMB, clinically relevant non major bleeding; VTE, venous thromboembolism; CHD, congenital heart disease; CVL, central venous line.

#### 3.2.2 Edoxaban for prevention of blood vessels being blocked by clots (thrombotic events) in children at risk because of cardiac disease (ENNOBLE-ATE trial published in 2022)

This was a phase 3, multinational, prospective, randomized, open-label, blinded trial comparing once-daily edoxaban to SOC, which included LMWH or VKA (20, 39). Edoxaban was administered as weight-adjusted doses in tablet or granule form to achieve adult-level exposures. Please refer to Table 1 for summary on efficacy and safety outcomes. It was concluded that edoxaban is an appropriate alternative for thromboprophylaxis in pediatric heart disease with the advantage of once-daily dosing**.**

#### 3.2.3 Rivaroxaban, a direct factor Xa inhibitor, versus acetylsalicylic acid as thromboprophylaxis in children post–fontan procedure: rationale and design of a prospective, randomized trial (the UNIVERSE study published in 2021)

This was a prospective, randomized, multicenter, 2-part, open-label trial comparing a novel liquid rivaroxaban formulation to aspirin (ASA) for primary thromboprophylaxis in children following the Fontan procedure (19, 40). Part A of the trial evaluated PK and pharmacodynamic properties of rivaroxaban to confirm dosing regimen used in part B. The rivaroxaban dosing regimen was designed to match adult exposures for a 10 mg daily dose, and PK modeling confirmed these targets were achieved. Please refer to Table 1 for summary on efficacy and safety outcomes in part B of the study. The trial concluded that rivaroxaban has an adequate safety and efficacy profile for children after the Fontan procedure.

#### 3.2.4 Dabigatran for treatment and secondary prevention of VTE in pediatric CHD (DIVERSITY *post-hoc* CHD subgroup analysis published in 2024)

The DIVERSITY study was a randomized, multicenter, open-label, phase 2b/3 trial evaluating dabigatran etexilate against SOC (heparins or VKAs) for the treatment of acute VTE (41). A *post-hoc* subgroup analysis was conducted on 48 children with CHD, of whom 21 received dabigatran. Please refer to Table 1 for summary on efficacy and safety outcomes. In a subsequent single-arm extension study, no recurrent VTEs were observed in the 17 CHD patients treated with dabigatran for up to 12 months. Dabigatran was dosed using an age- and weight-adjusted nomogram to achieve adult-equivalent exposures. This exploratory study concluded that dabigatran could be an appropriate treatment choice for children with CHD, demonstrating efficacy and safety consistent with the overall trial population.

#### 3.2.5 Pharmacokinetics of a microdosed cocktail of three direct oral anticoagulants in children with congenital heart defects (DOAC-child) (42)

This is a single-center, open-label, single-dose clinical trial designed to evaluate the PK and safety of three DOACs in pediatric patients with non-cyanotic CHD utilizing a microdose-cocktail [containing very low doses of apixaban (12.5 µg), rivaroxaban (12.5 µg), and edoxaban (50 µg), along with microdosed probe drugs]. Ultimately, the goal of this study is to facilitate the selection of the DOAC with the most favorable profile for further clinical research and pave the way for standardized pediatric anticoagulation guidelines. The study protocol was published in 2023; however, the trial results are not yet published.

Collectively, these studies have demonstrated that pediatric weight-adjusted dosing achieves drug exposure comparable to those observed in adults and have established an emerging evidence base supporting the use of DOACs for thromboprophylaxis and treatment of VTE in selected pediatric cardiac populations. However, it is noteworthy that none of these trials discussed above were statistically powered to evaluate efficacy clinical endpoints.

### 3.3 Systematic reviews and meta-analyses of DOAC clinical trials in pediatric cardiac populations

As evidence supporting the use of DOACs in pediatric CAHD has expanded, several systematic reviews and meta-analyses have synthesized the available clinical trial data. Collectively, these studies consistently demonstrate that DOACs provide efficacy and safety comparable to conventional anticoagulation while offering important practical advantages, including fixed weight-based dosing, elimination of routine laboratory monitoring, and improved ease of administration.

In 2023, Giossi et al. conducted a comprehensive systematic review and meta-analysis of six randomized controlled trials (RCTs) to evaluate the efficacy and safety of DOACs in children for VTE treatment and/or prophylaxis in cardiac disease in 2023 (43). The analysis incorporated the UNIVERSE trial and preliminary data from the ENNOBLE-ATE and SAXOPHONE studies. For the treatment of pediatric VTE, DOACs significantly reduced the recurrence of TE compared to SOC without an accompanying increase in bleeding risk or serious adverse events. Among children receiving primary thromboprophylaxis for pediatric heart disease, DOACs were found to be at least comparable to SOC in both safety and efficacy. Although treatment discontinuation due to adverse events was more frequent among patients receiving DOACs for VTE treatment, this trend was not statistically significant in the cardiac subgroup. The authors concluded that DOACs represent a safe alternative to conventional anticoagulants but emphasized that the available evidence remains limited by relatively small study populations and requires confirmation through larger real-world studies.

Recognizing the unique thrombotic risk associated with pediatric heart disease, Hong et al. (44) subsequently performed a meta-analysis specifically evaluating DOACs for thromboprophylaxis in pediatric CAHD. Data was pooled from the UNIVERSE, ENNOBLE-ATE, and SAXOPHONE trials with total of 471 participants. This study reported zero mortality across both the DOAC and control groups. The analysis revealed that DOAC users experienced a non-significant reduction in both VTE occurrence (0.33% vs. 1.95%) and clinically relevant non-major bleeding [CRNMB] (2.00% vs. 3.90%) compared to SOC. No significant differences were observed in the incidence of major bleeding (0.67% vs. 0.64%), any bleeding, or serious adverse events between the treatment groups. The authors concluded that direct factor Xa inhibitors were at least as safe and effective as VKA or LMWH, while offering a more convenient and child-friendly prophylactic option for children with CAHD.

Another systematic review of three landmark prospective RCTs (UNIVERSE, ENNOBLE-ATE, and SAXOPHONE) along with a *post-hoc* CHD subgroup analysis of the DIVERSITY trial to assess DOACs in pediatric heart disease was published by Guan et al. in 2024 (45). The review concluded that DOACs are at least as effective as the traditional anticoagulants (aspirin, VKAs, or LMWHs) in preventing TE events in pediatric heart disease. Safety outcomes were similarly positive, with major and CRNMB rates remaining low and comparable across all treatment groups. However, the researchers noted that the DIVERSITY *post-hoc* CHD analysis reported a significantly higher rate of minor bleeding in the DOAC arm (14.3% vs. 3.7%). Overall, the authors concluded DOACs have the potential to simplify treatment protocols, reduce monitoring burden, and enhance adherence in this vulnerable population.

Most recently, a meta-analysis by Bakry et al. (46) synthesized data from four RCTs (DIVERSITY, UNIVERSE, SAXOPHONE, and ENNOBLE-ATE) involving 732 pediatric patients with CAHD to provide a comprehensive perspective on DOAC safety and efficacy in this population. More importantly, this study included a prespecified CHD subgroup analysis that included patients from DIVERSITY *post-hoc* CHD subgroup and UNIVERSE trial, which significantly added to the strength of this metanalysis. Their results demonstrated that DOACs significantly reduced overall TE events compared to SOC (RR = 0.42, 95% CI: 0.18–0.97; *p* = 0.04). In the specific CHD subgroup, while the reduction in individual TE followed a similar trend but did not reach statistical significance, the pooled analysis for the composite efficacy endpoint showed a statistically significant benefit for DOACs (RR = 0.33, 95% CI: 0.13–0.87, *p* = 0.02). Consistent with prior research, no significant differences were observed in the rates of major bleeding, CRNMB, or serious adverse events between groups. The authors concluded that DOACs offer effective and safe thromboprophylaxis by reducing thrombotic risk without increasing bleeding complications, positioning them as an attractive alternative for long-term management in vulnerable pediatric populations where monitoring and adherence are particularly challenging. By isolating the CHD population, the work of Bakry et al. substantially advances the broader findings of Giossi, Hong, and Guan, offering targeted, high-quality evidence that supports the utility of DOACs in this highly specific and vulnerable pediatric cohort.

Taken together, these systematic reviews reinforce the findings of the individual clinical trials and support the growing role of DOACs as an alternative to conventional anticoagulation in selected pediatric cardiac populations. Across multiple studies, DOACs have consistently demonstrated comparable efficacy for thromboprophylaxis and VTE treatment, with no increase in major bleeding or serious adverse events. However, several important limitations remain. The rarity of pediatric cardiac thrombosis has resulted in relatively small sample sizes, low event rates, and heterogeneous study populations that limit statistical power for detecting differences in clinical outcomes. Furthermore, long-term safety data - including the effects of chronic anticoagulation on growth, neurodevelopment, bone health, hepatic function, and quality of life - remain limited. Additional multicenter prospective studies and post-marketing registries will therefore be essential to define optimal patient selection, lesion-specific treatment strategies, and the long-term role of DOACs in pediatric CHD.

### 3.4 Real-world experience and observational data

Although RCTs have established the safety and efficacy of DOACs in selected pediatric cardiac populations, real-world observational studies provide important insights into their effectiveness in routine clinical practice, where patients often present with greater clinical complexity, broader indications, and less stringent treatment protocols than those enrolled in clinical trials.

In the largest series to date, VanderPluym et al. (47) evaluated 219 pediatric patients with CAHD treated with apixaban. Overall, apixaban demonstrated a favorable safety profile, with a low incidence of major bleeding or CRNMB (2.9 per 100 patient-years). Among patients receiving apixaban for primary thromboprophylaxis, no new TE were observed, while 95% of those treated for active thrombosis achieved complete thrombus resolution. Importantly, more than half of the cohort received apixaban as a first-line therapy without the initial parenteral anticoagulation bridge traditionally required in pediatric DOAC clinical trials, suggesting increasing clinician confidence in the use of apixaban as first line in selected patients. This study also provides valuable practical guidance regarding therapeutic monitoring. Although routine laboratory monitoring is not recommended for apixaban by current regulatory guidance, the authors incorporated an institutional protocol for monitoring apixaban-specific anti-Xa peak levels during treatment initiation, new hemorrhagic or thrombotic events, and in the perioperative period. Dose adjustments were required in 25% of the cohort based on these levels, and thus selective monitoring may be considered in selected high-risk patients, although evidence remains insufficient to recommend routine monitoring.

Real-world evidence evaluating rivaroxaban in children with CAHD remains limited. Duran et al. (48) demonstrated the feasibility and overall safety of rivaroxaban in a single-center cohort of 27 children with CHD. However, a larger prospective observational study of 125 patients by Derrid et al. (49) reported a higher incidence of bleeding and thrombosis compared to RCTs data. Notably, heavy menstrual bleeding (HMB) emerged as an important complication among adolescent females receiving rivaroxaban, emphasizing the importance of individualized anticoagulant selection and proactive menstrual management in this population. Table 2 summarizes the published real-world evidence regarding DOAC use in pediatric CAHD.

**Table 2:** Real-world evidence of DOAC use in pediatric cardiac populations.

Publication (author, year)	DOAC studied	Target population	Population characteristics	Neonates and infants included	Treatment duration	Thrombotic rate (efficacy)	Bleeding rate (safety)
VanderPluym et al. (2023) (47)	Apixaban	CAHD requiring thromboprophylaxis or thrombosis treatment	*n* = 219 Median age 6.8 years	16 patients (7%) were younger than 1 year	Median 162 days (range 4–1217 days)	95% improvement of thrombosis in the treatment subgroup; 0% new events in the prophylaxis group.	2.9 per 100 pt-years (combined Major and CRNMB).
Dummer et al. (2023) (63)	Apixaban, dabigatran, rivaroxaban	Children and adults with giant coronary artery aneurysm due to Kawasaki disease	*n* = 24 (6 children) Median age 33.8 years	None	Median 4.9 years (IQR 4.2–8.0)	3 events of coronary thrombosis	No major bleeding.
Adkins et al. (2024) (55)	Apixaban	Post-Fontan procedure requiring thromboprophylaxis	*n* = 62 Median age 3.2 years	None	Median of 93 days (range 7–1,421 days)	1 event (1.6%), line related thrombosis	1.6% Major; 3.2% CRNMB; 9.7% any bleeding.
Duran et al. (2024) (48)	Rivaroxaban	CHD requiring thromboprophylaxis or thrombosis treatment	*n* = 27 Age range: 4 months-15 years	8 patients were younger than 1 year, no neonates included		83% resolution or stable thrombus (10/12); 0% new events in the prophylaxis group.	13.3 per 100 patient-years (2 clinically relevant episodes).
Benvenuto et al. (2024) (64)	Apixaban	Children awaiting heart transplant	*n* = 19 patient Median age 13.5 years	None	Median 114.6 days (range 38.5–174.5).	0% major thrombotic events while on heart transplant waitlist.	0% Major or CRNMB while on waitlist.
Sagiv et al. (2024) (62)	Edoxaban	Children with large coronary artery aneurysm due to Kawasaki disease	*n* = 16 Median age 7 years	None	Median 48.8 months.	2 events of coronary thrombosis	0% Major or CRNMB
Avsar et al. (2025) (54)	Rivaroxaban	Post-Fontan procedure requiring thromboprophylaxis	*n* = 192 received rivaroxaban)	None	Mean 5.1 years	2.1% thrombotic events	0.5% Major, 5.7% minor bleeding
Derridj et al. (2026) (49)	Rivaroxaban	CHD requiring thromboprophylaxis or thrombosis treatment	*n* = 125 Median age 9.3 years	No information	Median 8.5 months (IQR: 3.9–13)	4.2% patient-years (4 thrombotic events)	13.9 per 100 patient-years (16 clinically relevant episodes).

DOAC, direct oral anticoagulant; CAHD, congenital and acquired heart disease; CRNMB, clinically relevant non major bleeding; CHD, congenital heart disease.

Despite these promising institutional outcomes of DOAC use in pediatric heart disease, a survey of European pediatric cardiologists by Ryan et al. (50) in 2025 revealed highly variable clinical practices for the treatment of thrombosis in children with CAHD with respect to indication, agents used, and treatment duration. Importantly, 93% of respondents agreed that dedicated, evidence-based guidelines are urgently needed to standardize the use of DOACs in this heterogeneous and high-risk cardiac population. These findings underscore the disconnect between the rapidly expanding evidence base supporting DOACs and the absence of comprehensive, population-specific recommendations to guide routine clinical practice.

## 4 Special populations

### 4.1 Fontan circulation

Children with Fontan circulation represent the highest-risk CHD population for thromboembolic complications, with reported thrombosis rates ranging from 17%–33% (6, 7, 51). The Fontan circulation promotes thrombosis through multiple mechanisms, including chronic venous stasis, endothelial dysfunction, prosthetic material exposure, and hypercoagulability related to Fontan-associated liver disease and hepatic venous congestion. These pathophysiologic abnormalities persist throughout life, making lifelong thromboprophylaxis an important consideration.

Despite initial concerns regarding use of DOACs in Fontan patients due to potential hepatic dysfunction, recent clinical trial data suggest they are a safe and effective alternative to standard therapy in this population. The UNIVERSE trial demonstrated that children randomized to rivaroxaban post-Fontan had a lower rate of TE events compared to ASA (2% vs. 9%), while maintaining a comparable safety profile. Similarly, in the SAXOPHONE trial, where 74% of participants had single-ventricle physiology, apixaban was well-tolerated with no TE reported and bleeding rates comparable to SOC.

The growing body of evidence extends beyond pediatric trials. Two recent meta-analyses (52, 53) evaluating safety and efficacy of DOACs, VKA, and ASA thromboprophylaxis in adults with Fontan circulation concluded that DOACs had a stronger antithrombotic effect compared to warfarin and ASA, without increased major bleeding.

Emerging real-world evidence further demonstrates the safety and efficacy of DOACs in pediatric patients following Fontan procedures, filling critical gaps left by highly selective clinical trials. Avsar et al. (54) conducted a retrospective single-center study compared rivaroxaban to warfarin as postoperative anticoagulation in 369 pediatric patients after an extracardiac Fontan procedure, demonstrating a non-significant reduction in TE in the rivaroxaban group while showing a superior safety profile with significantly lower rates of major bleeding, mortality, and treatment discontinuation. Additionally, Adkins et al. (55) reported on the use of apixaban for early thromboprophylaxis in 62 patients post-Fontan, identifying low rates of both bleeding and thrombosis, which positions apixaban as a safe alternative in the immediate postoperative setting. Despite these encouraging findings, there are currently no dedicated international clinical guidelines specifically addressing DOAC selection, dosing, and duration of therapy in children with Fontan circulation.

A specific cohort that further complicates anticoagulation management is patients with Fontan-associated liver disease (FALD), a chronic and progressive complication driven by systemic venous hypertension and hepatic venous congestion. FALD encompasses a spectrum of liver abnormalities, including hepatic fibrosis, cirrhosis, and a long-term risk for hepatocellular carcinoma (56). Anticoagulation in this population is exceptionally complex due to a multifactorial coagulopathy: decreased Protein C levels, elevated Factor VIII, enhanced platelet activation, increased thrombin generation, and impaired fibrinolysis in Fontan circulation contribute to an increased thrombotic risk from venous stasis. Simultaneously, patients who develop advanced FALD with portal hypertension, thrombocytopenia, or esophageal varices face an elevated bleeding risk, creating a challenging therapeutic paradox (7, 28). Regarding DOACs, their suitability in this population varies by agent: rivaroxaban is contraindicated in moderate hepatic impairment (Child-Pugh B), whereas apixaban does not demonstrate significantly increased exposure in mild-to-moderate hepatic impairment and may be used with caution in those settings; all DOACs lack safety data in severe hepatic impairment (Child-Pugh C) (57). Dabigatran has the lowest hepatic metabolism among approved DOACs, as it is not metabolized by CYP450 enzymes and undergoes approximately 80% renal clearance. While the DIVERSITY trial *post hoc* CHD subgroup analysis demonstrated favorable safety and efficacy of dabigatran for VTE treatment in pediatric patients with CHD, it has not been specifically studied for thromboprophylaxis in the Fontan population. Because patients with advanced FALD were generally excluded from pivotal clinical trials, there remains insufficient evidence to guide standardized DOAC use specifically in the subgroup with severe FALD, necessitating a highly individualized approach.

### 4.2 Kawasaki disease (KD) with giant coronary artery aneurysms (GCAAs)

The incidence of GCAAs (defined by arterial diameter ≥8 mm) is approximately 5% in children treated with high-dose intravenous immune globulin and ASA, compared to 20%–25% in untreated populations (6, 58, 59). These aneurysms carry a lifelong risk of arterial thrombosis, myocardial infarction, and death (6, 58, 59). While thromboprophylaxis significantly reduces events compared to no treatment, historical data for LMWH and warfarin still demonstrate 2.5-year thrombosis rates of 5.7% and 6.7%, respectively (60). Consequently, interest in DOACs has increased as a more practical long-term anticoagulation strategy.

Recent data on DOACs in this population are primarily from the ENNOBLE-ATE and SAXOPHONE trials. In the ENNOBLE-ATE study, where KD patients comprised 22% of the cohort, edoxaban was found to be a feasible alternative to SOC; however, 6% (2/33) of KD patients in the open-label extension period developed arterial events with myocardial infarction, highlighting the persistent thrombotic risk associated with giant aneurysms (20). The SAXOPHONE trial (apixaban), which included a 14% KD subgroup, found the drug was safe and well-tolerated with low bleeding rates, though it was not powered to prove efficacy in preventing arterial events (21). Reflecting this data, the 2024 AHA Scientific Statement on KD now recognizes DOACs as a reasonable alternative to warfarin or LMWH for thromboprophylaxis in children with GCAAs (61). Real-world data across pediatric (*n* = 16) (62) and adult (*n* = 24) (63) cohorts with KD-related GCAAs indicate that DOAC therapy (including edoxaban, dabigatran, rivaroxaban, and apixaban) maintains a favorable safety profile with no reported major bleeding events over extended follow up. Nevertheless, the occurrence of several thrombotic and ischemic cardiac events despite anticoagulation emphasizes that patients with GCAAs remain at substantial residual cardiovascular risk and require continued multidisciplinary surveillance.

### 4.3 Other cyanotic CHD

Evidence for DOACs in cyanotic CHD outside of Fontan circulation remains extremely limited. Patients with Eisenmenger syndrome and other cyanotic lesions exhibit complex hemostatic abnormalities characterized by simultaneous bleeding and thrombotic tendencies, making anticoagulant selection particularly challenging. Consequently, current guidelines generally do not recommend routine DOAC use in this population due to insufficient data regarding safety, efficacy, and optimal dosing (37). Additional prospective studies are needed before broader adoption can be recommended.

### 4.4 DOACs in children awaiting heart transplant

Data on the use of DOACs for children awaiting heart transplant are primarily derived from real-world evidence, as these high-acuity patients are largely excluded from clinical trials. The most comprehensive data comes from a retrospective single-center study by Benvenuto et al. of 19 children who received apixaban while awaiting cardiac transplant (64). This study demonstrated that weight-based apixaban therapy achieved zero thrombotic or major bleeding events prior to surgery. While 32% of patients experienced post-operative bleeding, none were attributed to apixaban. Apixaban was discontinued for a median of 23.2-hour pre-operatively with low residual levels prior to surgery, supporting the feasibility for use in this population. These findings suggest that apixaban may represent a practical alternative to warfarin-based anticoagulation in carefully selected transplant candidates. However, following the U.S. withdrawal of andexanet alfa in December 2025 due to an unfavorable risk-benefit profile (driven primarily by excess TE), no specific reversal agent for factor Xa inhibitors remains available in the US. Idarucizumab continues to provide targeted reversal for dabigatran. In the absence of a specific factor Xa inhibitor reversal agent approved for pediatric use, clinicians must rely on the off-label use of prothrombin complex concentrates for bleeding or urgent procedures, emphasizing the importance of careful perioperative planning (65, 66).

### 4.5 Mechanical valve and prosthetic materials

The use of DOACs is currently contraindicated in patients with mechanical prosthetic heart valves. This restriction is primarily driven by the results of the RE-ALIGN trial (67), a phase II randomized study that compared dabigatran with warfarin in patients who had undergone heart valve replacement. The trial was terminated prematurely after enrolling 252 patients due to significantly increased rates of both TE and major bleeding in the dabigatran arm compared to the warfarin group. Further evidence supporting this contraindication comes from a more recent trial evaluating apixaban in patients with mechanical aortic valves, which also demonstrated an increased rate of TE in the apixaban arm (68). Consequently, VKAs remain the only recommended oral anticoagulants for this population, as they provide superior protection against stroke and valve thrombosis with a more favorable safety profile in this specific context.

### 4.6 Neonates and infants

Neonates and young infants remain among the least studied populations in pediatric anticoagulation. Most clinical trials have excluded patients based on gestational age and weight, resulting in a substantial evidence gap (69). Anticoagulation management requires individualized dosing strategies to account for the dynamic physiological changes of childhood, including developmental hemostasis, where age-dependent levels of procoagulant and anticoagulant proteins do not reach adult concentrations until puberty (6). Infants and neonates exhibit a higher volume of distribution for many anticoagulants due to higher total body water content and demonstrate accelerated drug clearance, resulting in significantly shorter half-lives. Furthermore, the gradual maturation of hepatic metabolic pathways, particularly the CYP3A4 system which achieves 50% of adult capacity by late infancy, creates highly variable drug exposures that may be further modified by cardiac-related hepatic dysfunction (70) (45). Furthermore, standardized liquid formulations for age-appropriate dosing are lacking, creating a critical unmet need in treatment options.

While the SAXOPHONE trial was designed to include infants as young as 28 days, none included were under 4 months of age, leaving a critical data gap regarding early infancy. Consequently, current regulatory labeling for agents like rivaroxaban remains highly restrictive. For example, rivaroxaban is not recommended for preterm infants under 6 months of age, those with a body weight <2.6 kg, or infants who have not established full enteral feeding. While small real-world case series suggest DOACs may be effective in reducing thrombus burden in those under two years of age without unexpected safety concerns (71), larger prospective PK studies and clinical trials are urgently needed to establish standardized dosing, safety, and efficacy in this highly vulnerable patient population.

## 5 Practical recommendations for clinical use of DOACs in pediatric heart disease

For clinicians managing pediatric CAHD, DOACs offer a promising alternative for thromboprophylaxis in stable patients with Fontan circulation and Kawasaki disease with GCAAs, provided they have adequate organ function and can tolerate oral intake. Initiation typically follows weight-based nomograms using age-appropriate formulations, often after 5 days of parenteral anticoagulation bridge, though real-world practice is increasingly shifting toward first-line use without a bridge in selected cohorts. However, Rivaroxaban remains the only DOAC with a specific FDA-approved indication for thromboprophylaxis after the Fontan procedure in children aged ≥2 years and can be initiated directly without a parenteral anticoagulation bridge. Emerging data suggests potential efficacy for apixaban in children awaiting heart transplant, although data is limited to be able to provide recommendations in this patient group. While routine monitoring is not mandatory, measuring drug-specific anti-Xa levels might be considered in high-risk cardiac patients to guide dose adjustments, as developmental hemostasis and PK variability might necessitate dosing modifications (47). Importantly, DOACs remain contraindicated in patients with mechanical prosthetic valves or triple-positive antiphospholipid syndrome, and their use in neonates and preterm infants remains a “data-free zone” requiring extreme caution. For patients undergoing invasive procedures, DOACs should be interrupted 24 h before low-risk and 48 h before high-risk interventions to minimize bleeding complications. Despite fewer drug-drug interactions compared to VKA, clinicians should remain attentive to potential interactions, as rivaroxaban and apixaban are substrates of both CYP3A4 and P-glycoprotein. Of particular relevance in CAHD, antiarrhythmics (amiodarone, verapamil, and diltiazem) and cyclosporine (often used post-transplant) may result in increased DOACs concentration and thus possible increased risk of bleeding (72, 73). Table 3 provides summary for clinicians on the use of DOAC in pediatric CAHD. Ultimately, while real-world data across diverse pediatric cardiac cohorts continues to evolve, consolidated national or international guidelines are urgently needed to address remaining data gaps, guide clinical management, and standardize treatment protocols.

**Table 3:** DOAC Use in pediatric congenital and acquired heart disease (CAHD): summary for clinicians.

Cardiac condition	DOAC options	FDA approval	Weight-based dosing	Patient selection & clinical considerations	Monitoring
Acute VTE treatment & secondary prophylaxis	Dabigatran, Rivaroxaban, Apixaban	Yes	Age- and weight-adjusted nomograms or dosing regimens available	Approved for use after at least 5 days of initial parenteral anticoagulation (e.g., heparin-based).	Routine monitoring is not recommended
Fontan circulation	Rivaroxaban (FDA approved >2 yrs); Apixaban (studied)	Only For rivaroxaban >2 yrs	Weight-based adjusted dosing	Rivaroxaban demonstrated numerically lower TE rates compared to aspirin in the UNIVERSE trial. Concerns for HMB for rivaroxaban in adolescent females	Generally, no routine monitoring. Clinicians should be aware of potential Fontan-associated liver disease when selecting agents. Avoid in patients with severe hepatic impairment.
Kawasaki disease (KD) with GCAAs	Edoxaban (studied); Apixaban (studied)	None	Weight-adjusted doses (tablets or granules) to achieve adult-level exposures.	GCAAs carry a lifelong risk of arterial thrombosis. AHA now recognizes DOACs as a reasonable alternative to warfarin or LMWH.	Multidisciplinary surveillance is required as residual cardiovascular risk remains high despite anticoagulation.
Heart transplant candidates	Apixaban (real-world data)	None	Standard pediatric weight-based dosing.	Feasible for carefully selected candidates. Real-world study showed zero thrombotic or major bleeding events prior to surgery.	Discontinue ∼24 h pre-operatively. Consider apixaban-specific anti-Xa peak levels during treatment initiation, perioperative periods, or when managing new hemorrhagic/thrombotic events.
Other cyanotic CHD (e.g., Eisenmenger)	Not recommended	None	N/A	Insufficient safety and efficacy data; these patients exhibit complex simultaneous bleeding and thrombotic tendencies.	N/A
Mechanical heart disease	Not recommended	None	N/A	N/A	N/A
Neonates and infants (<6 months) with CAHD	Rivaroxaban (restricted); Apixaban (studied >28 days)	None	Carefully individualized dosing due to developmental changes in drug clearance and hepatic metabolism	Rivaroxaban is not recommended for preterm infants <6 months, weight <2.6 kg, or those not on full enteral feeding.	

DOAC, direct oral anticoagulant; VTE, venous thromboembolism; TE, thrombotic events; HMB, heavy menstrual bleeding; GCAAs, Giant coronary artery aneurysm; AHA, American Heart Association; LMWH, low molecular weight heparin; N/A, not applicable indicating no information available.

## 6 Knowledge gaps and future research directions

Despite the rapidly expanding evidence supporting DOACs in pediatric CAHD, several important knowledge gaps remain. Most prospective trials have demonstrated favorable short-term safety and efficacy; however, long-term outcomes in children and adolescents remain largely unknown. Unlike adults, pediatric patients undergo continuous growth and development, raising important questions regarding the potential effects of chronic DOAC exposure on skeletal maturation, neurodevelopment, organ development, and overall health across the lifespan.

A notable concern for adolescent females is HMB. Although HMB was not systematically evaluated in the landmark pediatric clinical trials, subsequent real-world studies have demonstrated substantially higher rates among adolescents receiving rivaroxaban, with reported incidences approaching 46% in some cohorts, compared with apixaban or conventional anticoagulation (74). These findings underscore the importance of incorporating menstrual outcomes into future pediatric anticoagulation studies and emphasize the need for individualized anticoagulant selection in adolescent females.

Similarly, the long-term effects of DOAC therapy on bone health remain uncertain. Prolonged exposure to traditional agents like warfarin and LMWH are known to negatively affect bone mineral density in adult populations, though pediatric-specific data are limited. Whether DOACs confer a skeletal advantage during the critical period of bone mineral acquisition in childhood and adolescence remains unknown, and it represents a high priority for future investigation (21, 45).

Beyond bone health, substantial data gaps persist regarding the effects of DOACs on complex physiological processes such as neurodevelopment, angiogenesis, and overall growth. Evidence remains insufficient regarding DOAC initiation at diagnosis without lead-in parenteral anticoagulation, pediatric-specific cost-effectiveness, and safety in complex subgroups, including preterm infants and patients with renal or hepatic failure or those with arterial thrombotic events. The scarcity of periprocedural guidelines and clinical data on reversal agents in children further highlights the need for targeted pediatric research.

Patient-reported outcomes represent another important area of investigation. Although adult registries like NOTE have documented significant quality of life (QoL) improvements in domains such as social functioning and mental health after transitioning to DOACs, these results cannot be directly extrapolated to the pediatric setting (75). Preliminary findings from the SAXOPHONE trial suggest reduced treatment-related anxiety and improved treatment convenience with apixaban, yet small sample sizes and exploratory analyses limit definitive conclusions. Consequently, prioritizing longitudinal research studying the impact of DOACs on pediatric QoL is essential to achieving patient-centered anticoagulation stewardship.

**Figure 2:**
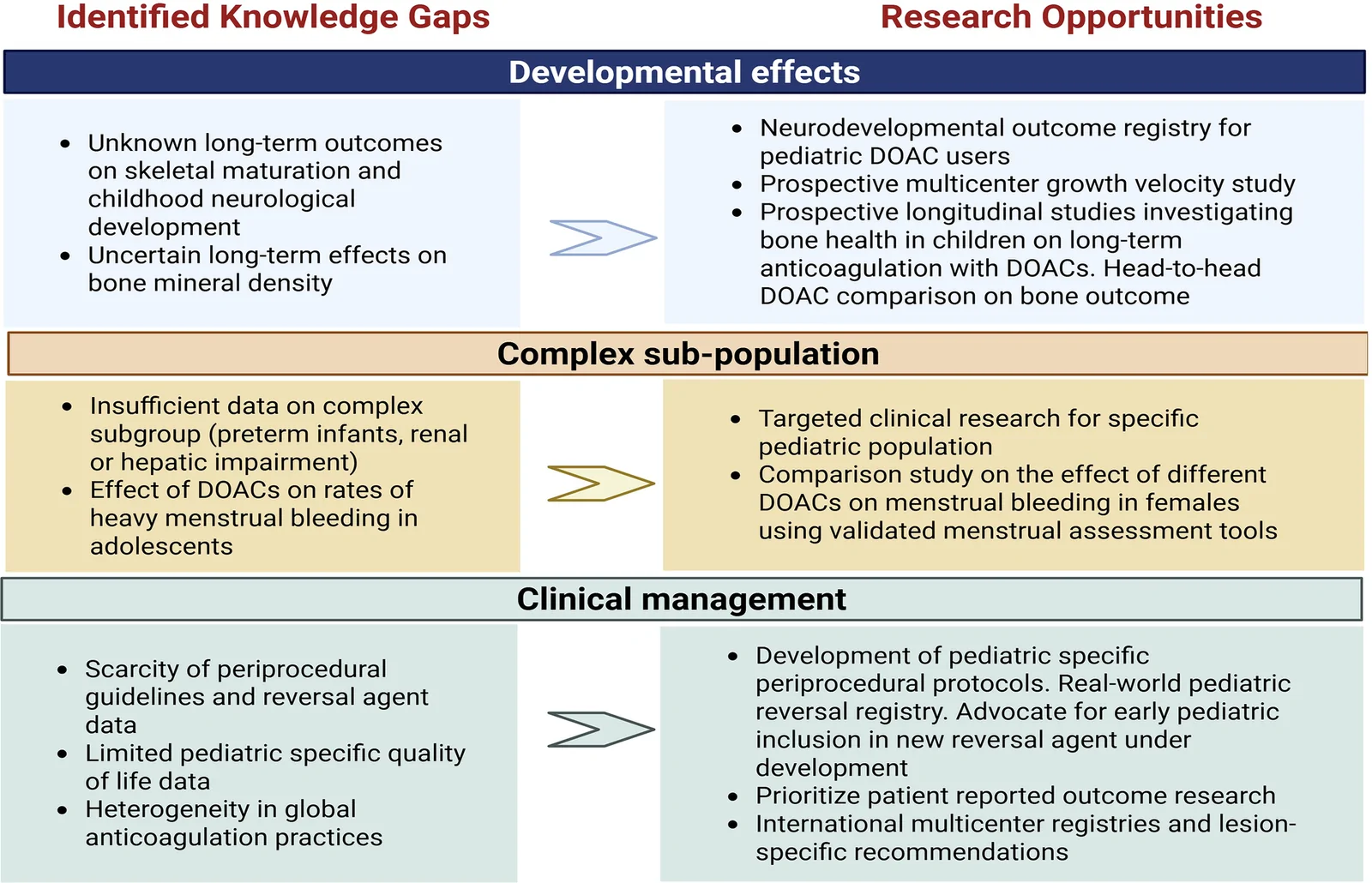
Strategic roadmap for DOAC research in pediatric CAHD: from knowledge gaps to research opportunities. This figure illustrates the identified evidence gaps to prioritized research objectives for DOACs in children.

Finally, although evidence supporting DOAC use continues to grow, significant heterogeneity persists in anticoagulation practices across institutions and countries. Figure 2 illustrates the major knowledge gap identified and research objectives to advance the use of DOACs in CAHD. International collaboration will be essential to establish prospective registries, perform adequately powered multicenter studies, and develop lesion-specific, evidence-based recommendations that address the unique needs of children with CHD.

## 7 Conclusion

The introduction of DOACs represents one of the most significant advances in pediatric anticoagulation over the past decade. Emerging evidence from RCTs, observational studies, and real-world experience consistently demonstrates that weight-adjusted DOAC therapy provides efficacy comparable to conventional anticoagulation while maintaining favorable safety profiles in selected children with CAHD. In addition to eliminating routine laboratory monitoring, DOACs offer practical advantages that have the potential to improve treatment adherence, reduce healthcare burden, and enhance quality of life for children and their families.

Despite these advances, a significant gap remains as updated international guidelines have yet to be established to standardize DOACs use in this population. Consequently, substantial variability persists regarding patient selection, anticoagulant choice, treatment duration, monitoring strategies, and peri-procedural management. Future research must prioritize inclusion of underrepresented groups like neonates, development of safe reversal agents, and the investigation of long-term impacts on quality of life, bone density, and neurodevelopment. Additionally, age-appropriate liquid formulations must become widely available and accessible for patients.

As the field continues to evolve, collaboration among pediatric cardiologists, hematologists, thrombosis specialists, pharmacists, and regulatory agencies will be critical to generating the high-quality evidence needed to optimize anticoagulation across the spectrum of pediatric heart disease. With continued multicenter research and thoughtful integration of emerging data, DOACs have the potential to meaningfully advance thromboprophylaxis in this population, offering a more practical alternative to traditional anticoagulants with a growing evidence of comparable efficacy and improved tolerability.
